# The contributions of lean muscle, intramuscular fat, and subcutaneous fat to lipids within the beef matrix

**DOI:** 10.1093/jas/skaf450

**Published:** 2025-12-29

**Authors:** Kyra L Elliott, Tate S Johnson, Jessie C Morrill

**Affiliations:** Department of Animal Science, University of Nebraska—Lincoln, Lincoln, NE 68503; Department of Animal Science, University of Nebraska—Lincoln, Lincoln, NE 68503; Department of Animal Science, University of Nebraska—Lincoln, Lincoln, NE 68503

**Keywords:** beef matrix, fat, lipidomics, meat, muscle, nutrition

## Abstract

Incidence of obesity and associated metabolic diseases is increasing at a rapid rate, with one in three Americans diagnosed as obese. This creates an urgent need for an understanding of the nutritional value of foods and the roles they play in the development of obesity and metabolic diseases. When evaluating the role of beef in the diet, all of beef’s nutritive and non-nutritive components should be considered as part of a whole food beef matrix. A serving of beef may contain multiple tissue types including lean muscle (LM), intramuscular fat (IMF), and subcutaneous fat (SF). As these are metabolically unique tissues, we hypothesize that these tissues contribute unique lipids to the beef matrix. To test the hypothesis, LM, IMF, and SF were dissected from five strip loins from cattle finished using a standard feedlot diet. The lipidome of the tissues was then determined using shotgun lipidomics. Each tissue type had a distinct lipid signature and contained different proportions of storage lipids, membrane lipids, and lysolipids (*P *< 0.01). Notably, LM contained fewer storage lipids and higher proportions of membrane lipids and lysolipids compared to IMF and SF, whereas IMF and SF did not differ significantly from each other. While most triacylglycerol (TAG) classes were similar across all tissues, SF contained greater proportions of TAG with 53, 55, and 56 carbons than LM and IMF (*P *≤ 0.03). Across all tissues, TAG with 52 carbons and 2 double bonds was the most abundant. Membrane lipid composition of SF contained higher proportions of phosphatidylcholine, phosphatidylglycerol, phosphatidylserine, and ceramide than LM and IMF (*P *≤ 0.03). Proportion of diacylglycerol in IMF and SF membrane lipids was higher compared to LM (*P *< 0.01). Lean muscle contained higher proportions of ether-linked phospholipids than IMF and SF (*P *< 0.01). Lysolipids make up ≤ 1% of total lipids in LM, IMF, and SF; LM contains a higher proportion of lysolipids than IMF or SF (*P *< 0.01). Of the lysolipids. Of the lysolipids, lysophosphatidylcholine (LPC) is the most abundant across all tissue types; IMF contains higher proportions of LPC compared to LM and SF (*P *< 0.01). These data provide insight into the lipid composition of the whole food beef matrix of beef strip steaks and suggest that the whole food beef matrix may be influenced by fat trim level (amount of SF) and IMF content.

## Teaser

Understanding the lipids that are present in beef is key to understanding the role of beef in a healthy diet. The proportions of lean muscle, intramuscular fat, and subcutaneous fat in a serving of beef may have important implications on the nutritional value of beef.

## Graphical abstract

**Graphical Abstract:**
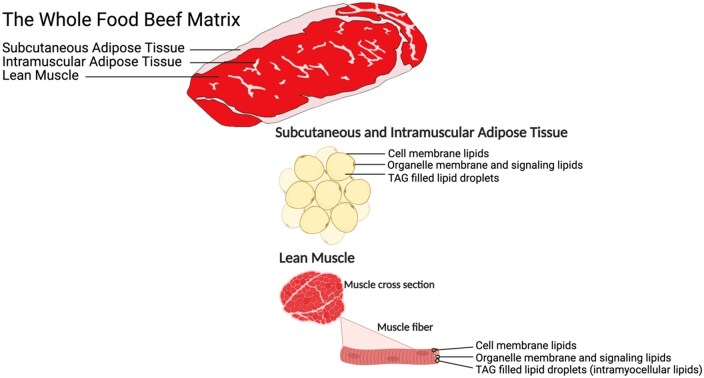

## Introduction

In August 2021, the National Cattlemen’s Beef Association convened a meeting that was attended by nutrition science experts, during which the term “beef matrix” was defined as: “*the collective nutritive and non-nutritive components of the beef food structure and their unique chemical and physical interactions that may be important for human health and are distinguishable from those of the single components in isolation*” (Klurfeld, 2024). Food matrices are a relatively new concept in nutrition science, and the beef matrix, in particular, is not yet fully characterized. The beef matrix is likely to be complex: animal management practices, harvest conditions, cut of beef, conditions of aging, packaging methods, amount and type of further processing, and cooking techniques are all likely to influence both the nutritive and non-nutritive components of the matrix. A better understanding of how these factors impact the beef matrix could improve how we evaluate beef in the context of human health.

Gaps in knowledge about the beef matrix were identified by attendees of the meeting and were reported by Klurfeld (2024). An understanding of the nutritional and structural profile of the beef matrix as well the chemical diversity and structure/function relationships within beef tissues was identified as key research needs.

Prior research has shown that subcutaneous fat from various depots throughout the beef carcasses can differ in fatty acid composition (Turk and Smith, 2009), others have shown that there are also notable differences in fatty acid composition between subcutaneous fat and intramuscular fat (Sturdivant et al., 1992; May et al., 1993; Archibeque et al., 2005). While these studies have uncovered differences in fatty acid composition among adipose tissue depots, the contributions of subcutaneous fat, intramuscular fat, and lean muscle to the overall lipidome of the beef matrix are still unknown. Among beef retail cuts, there is fluctuation in the ratios of subcutaneous fat, intramuscular fat, and lean muscle. Thus, the types and amounts of lipids within a retail cut is subject to change and may impact the beef matrix. To address gaps in the understanding of the beef lipidome and its contributions to the beef matrix, we conducted a study to elucidate an understanding of the lipid composition of intramuscular fat, subcutaneous fat, and lean muscle present in beef steaks using shotgun lipidomics.

Shotgun lipidomics is an emerging technology that allows for sensitive and detailed quantification of lipid compositions of biological samples. To date, the application of lipidomics among meat products has been primarily for detecting food adulteration and fraud (Harlina et al., 2022). Lipidomics has proven to be a useful technology in this regard because animal species (i.e., pigs, and cattle) have unique lipidomes (Trivedi et al., 2016). To our knowledge, shotgun lipidomics has not been utilized to characterize the distinct lipidomes of beef subcutaneous fat, intramuscular fat, or lean muscle. We hypothesize that these tissues have distinct lipidomes that serve both structural and functional roles and thus contribute different lipids to the beef matrix.

The primary objective of this study was to utilize shotgun lipidomics to characterize the lipids present in the lean muscle, intramuscular adipose tissue, and subcutaneous adipose tissue of beef strip steaks. Determining the lipid compositions of each of these tissues will provide better insight into their respective contributions to the nutritive components of the beef matrix and the overall lipid profile of beef.

## Materials and Methods

### Study approval

The experimental protocol for the cattle feeding portion of the experiment was approved by the Institutional Animal Care and Use Committee at the University of Nebraska—Lincoln (protocol # 2226).

### Animals and beef sample procurement

Five untrimmed beef strip loins (Institutional Meat Purchase Specification 180; NAMP, 2011) were collected from carcasses that qualified for the Certified Angus Beef program at a commercial beef packing facility. Since comparable lipidomics studies have not previously been performed using the beef tissue types of interest, we conducted a power analysis using the pwr.anova.test function in R (version 4.0) and R Studio (pwr 1.3-0) using estimates of means and standard deviations from tissue culture and rodent studies in the literature (Levental et al., 2020). The power analysis for three tissue types (*k* = 3), with an effect size *f *= 1, alpha = 0.05 and desired power = 0.8 indicated 4.38 samples were required per tissue type; thus, five samples per tissue type were sought. To reduce variation among samples, the carcasses that were sampled were obtained from steers fed a standard finishing diet at the Eastern Nebraska Research, Extension and Education Center (ENREEC) near Mead, NE from June to December 2024 (Table 1). The carcasses utilized were from control cattle from a feedlot trial unrelated to this article. At the conclusion of the feeding period, cattle were transported to a commercial beef packing facility, and animal identification was maintained throughout harvest, chilling, and grading. After approximately 46 h of chill, ribeye area, backfat thickness, and marbling score were measured using a commercial beef grading camera (Table 2). After grading, loins were selected for collection. All loins were from unique pens of cattle so that loin could serve as the experimental unit. Collected loins were then vacuum packaged and transported to our laboratory.

**Table 1.** Composition of finishing diet provided to steers for approximately 150 days prior to harvest^1^

Item	Finishing diet
**Ingredient, % of diet on a dry basis**	
**High moisture corn**	56
**Cargill sweet bran**	18
**MDGS^2^**	12
**Corn silage**	7
**Corn stalks**	3
**Supplement^3^**	4

1 All steers were implanted with a Rev-XS upon entry into the feedlot.

2 MDGS, modified distiller grains plus solubles.

3 Supplement (UNL BN-2304) contains finely ground corn meal as a carrier and provides Rumensin (30 g/ton of DM), Tylan (8.8 g/ton of DM), minerals and vitamins to meet or exceed nutrient requirements, and urea (0.6% DM) for rumen degradable protein needs. For the final 28 days of the finishing period, Optaflexx was included in the supplement.

**Table 2.** Summary statistics for carcass characteristics and objective color values of beef strip loins utilized for sample collection for lipidomics analysis

Item	Mean	SD^1^
**Carcass characteristics**		
**Hot carcass weight**	432	17.3
**Ribeye area, cm^2^**	98.4	5.51
**Fat thickness, cm**	1.8	0.41
**Marbling score^2^**	609	43.3
**Objective color**		
L*	41.6	2.41
a*	21.0	1.68
b*	12.0	1.46

1 SD, standard deviation.

2 400 = small, 500 = modest, 600 = moderate.

### Lipidomics sample preparation and analysis

Immediately upon arrival to our laboratory, strip loin packages were opened and lean muscle tissue (LM), intramuscular adipose tissue (IMF), and subcutaneous adipose tissue (SF) were dissected from the anterior face of the loin. To ensure repeatability of our procedures, LM was sampled at a location approximately three-fourths the length of the loin eye, adjacent to the region of preliminary yield grade measurements, in the approximate center of the muscle. The LM sample was approximately 1 cm^3^ and was carefully cleaned with a scalpel to be devoid of any contaminating adipose tissue. This anatomic location was selected to ensure consistency and repeatability. Likewise, several intramuscular adipose tissue (IMF) pieces were dissected from the face of the loin eye using a scalpel and were also carefully cleaned to be devoid of contaminating muscle. Subcutaneous adipose tissue was dissected from a region approximately three-fourths up the length of the loin eye, for ease and repeatability between loins and future experiments. Total wet tissue weight required for lipidomics analysis is 1.5 mg per sample (Lipotype GMBH submissions require 300ul of homogenized tissue at a concentration of 5 mg wet tissue/mL), so large quantities of each tissue type were not sought.

Tissue samples were snap frozen in liquid nitrogen and were stored at −80°C until preparation for lipidomics. In preparation for lipidomics analysis, guidelines from Lipotype GmbH (Dresden, Germany) were followed. Briefly, samples were homogenized using a metal bead homogenizer (TissueLyser LT; Qiagen; Germantown, MD) in either water (LM) or ethanol/water 1:1 (v/v; IMF and SF) and were diluted serially to achieve a final concentration of 5 mg/mL (wet tissue weight per volume). Samples were shipped on dry ice to Lipotype GmbH (Dresden, Germany) for shotgun lipidomics analysis, which was performed as previously described (Levental et al., 2020).

Upon arrival to Lipotype GmbH, samples were stored at −80°C until analysis. Prior to analysis, samples were thawed at 4°C. Lipotype GmbH conducted an exploratory experiment to determine sample concentration and further diluted samples, if necessary. On average, 150, 380, and 75ul of sample were analyzed for lean muscle, subcutaneous adipose tissue, and intramuscular adipose tissue, respectively.

Briefly, samples were spiked with lipid class-specific internal standards to assure absolute quantification of lipids. Lipids were extracted using an automated chloroform: methanol extraction procedure using a Hamilton Robotics STARlet (Reno, NV; Surma et al., 2021). Sample infusion was performed with a nanoelectrospray ionization technology (TriVersa NanoMate; Advion Interchim Scientific; Ithaca, NY) and mass spectrometry was performed with a Thermo Scientific Q Exactive Orbitrap (Waltham, MA). LipotypeXplorer, a proprietary software developed by Lipotype GmbH, was used for identification of lipids in the mass spectra. Further data processing and analyses were performed using the Lipotype laboratory information and management system and LipotypeZoom—a web browser-based data visualization tool.

The report provided from Lipotype GmbH indicated that sample processing went without any incidents and the ionization quality was satisfactory. The report stated that signal-to-noise ratio was in the range of hundreds and more, indicating high quality spectra. Lipotype GmbH included mammalian full blood quality control reference samples for cholesterol esters (CE), ceramide (Cer), diacylglycerol (DAG), hexosylceramide (HexCer) lysophosphatidic acid (LPA), lysophosphatidylcholine (LPC), ether-linked lysophosphatidylcholine (LPC O-), lysophosphatidylethanolamine (LPE), ether-linked lysophosphatidylethanolamine (LPE O-), lysophosphatidylinositol (LPI), phosphatidic acid (PA), phosphatidylcholine (PC), ether-linked phosphatidylcholine (PC O-), phosphatidylethanolamine (PE), ether-linked phosphatidylethanolamine (PE O-), phosphatidylinositol (PI), phosphatidylserine (PS), sphingomyelin (SM), and triacylglycerol (TAG) in the same analytical run to assess technical reproducibility. The median coefficient of variation across all classes was reported to be 10.9%. Blank samples were also included in the same batch and did not contain any unusual background.

In our laboratory, quantification of lipids was performed similarly to Levental et al. (2020). Briefly, data were first processed by transforming all lipids into mol% of total lipids extracted/detected. Then, the datasets were broken down further into functional category, class, and species compositions. In some cases, the distribution and structural characteristics (e.g., number of carbons or unsaturations in the acyl chains) of the individual species were quantified. All calculations were performed using R (version 4.0) and R Studio.

The nomenclatures used to describe lipids are aligned with that of Lipotype GmbH, the shorthand notation for MS-derived lipid structures (Liebisch et al., 2020; Kopczynski et al., 2022) and the SwissLipids database (Aimo et al., 2015) for unambiguous identification of lipid species.

### Loin color evaluation

As part of our laboratory’s routine loin collection procedures, instrumental color attributes (lightness (L*), redness (a*), and yellowness (b*)) of a 2.54 cm thick steak cut from the anterior portion of the loin were measured in the bloomed condition according to AMSA guidelines (King et al., 2023). Measurements were obtained using a calibrated chromameter set to the D65 illuminant (CR-400; Konica Minolta; Ramsey, NJ), that provided Commission Internationale de l’Éclairage (CIE) color space values (L*, a*, and b*). Color attributes were assessed in three locations, and the average L*, a*, and b* values were used to determine the summary statistics. Data are provided in Table 2 to document that loins were free from dark-cutting or other color abnormalities.

### Statistical analysis

Data were analyzed statistically using R (version 4.0) and RStudio (tidyverse version 1.3.0, lme4 version 1.1, emmeans version 1.8.0). For all analyses, loin/animal was considered the experimental unit. The fixed effect was considered significant at *P *≤ 0.05. Pairwise comparisons were performed using the emmeans() function with the Tukey’s adjustment and were considered significant at *P *≤ 0.05.

## Results and Discussion

The three broad functional categories of lipids found in cells and tissues are storage lipids, membrane lipids, and lysolipids. Storage lipids include triacylglycerols (TAG) and sterol esters. As a percentage of total extracted lipids in the beef samples that were analyzed, storage lipids represent the most abundant functional lipid category. Intramuscular fat and SF contain much higher proportions of storage lipids compared to LM (>96% vs. 68%; Table 3; *P *< 0.01).

**Table 3.** Proportions of functional lipids in beef lean muscle, intramuscular fat, and subcutaneous fat^1^

Item	LM^2^	IMF^3^	SF^4^	SEM^5^	*P*-value
**Functional Category, % of total extracted lipids**					
**Storage lipids^6^**	68.6^a^	98.7^b^	96.5^b^	3.28	<0.01
**Membrane lipids^7^**	30.4^a^	1.2^b^	3.2^b^	3.20	<0.01
**Lysolipids^8^**	1.01^a^	0.06^b^	0.34^b^	0.100	<0.01

1 Within each row, means with differing superscripts differ (*P* ≤ 0.05).

2 LM, lean muscle.

3 IMF, intramuscular fat.

4 SF, subcutaneous fat.

5 SEM, standard error of the mean.

6 Storage lipids includes triacylglycerols and sterol esters.

7 Membrane lipids includes ceramide, hexosylceramide, cardiolipin, diacylglycerol, phosphatidic acid, phosphatidylcholine, ether-linked phosphatidylcholine, phosphatidylethanolamine, ether-linked phosphatidylethanolamine, phosphatidylglycerol, phosphatidylinositol, phosphatidylserine, and sphingomyelin.

8 Lysolipids includes lysophosphatidic acid, lysophosphatidylcholine, ether-linked lysophosphatidylcholine, lysophosphatidylethanolamine, ether-linked lysophosphatidylethanolamine, lysophosphatidylinositol, lysophosphatidylglycerol, and lysophosphatidylserine.

The storage lipids in LM are most likely affiliated with intramyocellular lipid droplets, or lipid droplets contained within muscle fibers. While little is known about intramyocellular lipids in bovine tissues, it has been suggested that intramyocellular lipid deposition is related to enhanced marbling deposition in cattle (Jeong et al., 2012). This relationship is thought to be related to expression and activity of glycerol-3-phosphate acyltransferase (GPAT1; Newson et al., 2011; Jeong et al., 2012), which initiates triglyceride synthesis. Jeong et al., 2012) demonstrated that GPAT1 mRNA abundance in longissimus muscle was strongly and positively correlated with intramuscular fat content in Korean steers. It is not currently known how the amount of intramyocellular lipid, in the form of TAG, impacts the whole food beef matrix and thus, the nutritional value of beef.

While storage lipids are the most abundant lipid category in LM, LM has a significantly higher proportion of membrane lipids compared to IMF or SF (*P *< 0.01). Membrane lipids consist of ceramide (Cer), hexosylceramide (HexCer), cardiolipin (CL), diacylglycerol (DAG), phosphatidic acid (PA), phosphatidylcholine (PC), ether-linked phosphatidylcholine (PC O-), phosphatidylethanolamine (PE), ether-linked phosphatidylethanolamine (PE O-), phosphatidylglycerol (PG), phosphatidylinositol (PI), phosphatidylserine (PS), and sphingomyelin (SM).

Klurfeld (2024) indicated that adipocytes have phospholipid-enriched cell membranes that surround the lipids that are consumed as fat in meat. Our results demonstrate that LM will contribute a greater proportion of phospholipids than adipose tissue to the whole food beef matrix. Our results indicate membrane lipids account for only 30.4% of the total lipids in LM whereas storage lipids comprise 68% of the total lipids in LM. Thus, LM will be a major contributor of TAG to the whole food beef matrix, even in lean cuts of beef. As indicated by Savell et al. (1986), longissimus muscle that is practically devoid of marbling has 1.77% ether extractable lipid. Our results would suggest that a majority of this extractable lipid in lean beef could be from TAG within muscle fibers.

Lean muscle also contains a significantly higher proportion of lysolipids than IMF or SF (*P *< 0.01). Lysolipids predominantly function as signaling lipids, which mediate cellular processes related to membrane dynamics, inflammation, immune responses, and cell proliferation. The greater proportion of lysolipids observed in LM may reflect enhanced membrane turnover and metabolic activity characteristic of myocytes compared with adipocytes (Pradas et al., 2018). Elevated levels of LPC and LPE may indicate increased phospholipid remodeling, which could influence membrane fluidity, intracellular signaling, and susceptibility to oxidative stress. Collectively, these findings suggest that the phospholipid composition of lean muscle is structurally distinct from that of adipose depots and may contribute to cellular processes influencing muscle function and meat quality. Lysolipids that were quantified by shotgun lipidomics include: lysophosphatidic acid (LPA), lysophosphatidylcholine (LPC), ether-linked lysophosphatidylcholine (LPC O-), lysophosphatidylethanolamine (LPE), ether-linked lysophosphatidylethanolamine (LPE O-), lysophosphatidylinositol (LPI), lysophosphatidylglycerol (LPG), and lysophosphatidylserine (LPS).

In a recent study that evaluated the nutritional adequacy of vegan diets, it was found that a vegan meal plan only provided 162.8 mg choline, or 30% of the daily recommended value for choline (Graham et al., 2023). Choline is an essential nutrient that is important for neurodevelopment as well as cholinergic neurotransmitter signaling. Insufficient choline intake has been shown to have negative implications on cognitive function in aging adults (Liu et al., 2021). In clinical trials, soy-derived lysolecithin has been shown to increase blood choline concentration (Tanaka-Kanegae et al., 2024a, 2024b). Similarly, in a biomedical model, egg-derived and soy-derived LPC have mitigated acetylcholine depletion in the brain (Tanaka-Kanegae et al., 2023). Wallace and Fulgoni (2017) indicated that consumers of eggs have choline intake that aligns with recommended intake levels. Additionally, they found that consumers of meat, poultry, and seafood have increased levels of choline intake compared to those that do not eat meat, poultry, or seafood, but intake levels of choline are still not within the ideal range. Thus, there is room to improve the nutritional value of beef, by increasing its LPC content. Strategies to increase LPC content in a serving of beef, such as through reducing its fat content, warrants further consideration.

Subcutaneous fat contained greater proportions of TAG with 53, 55, and 56 carbons compared to LM and IMF (*P *≤ 0.03; Table 4). Proportions of TAG containing 40–52, 54, or 58 carbons did not differ across tissues (*P *≥ 0.11). Interestingly, a higher proportion of TAG within IMF contained one double bond (*P *= 0.05) whereas a higher proportion of TAG within LM contained 2 double bonds (*P *= 0.03). While not compared statistically, SF had numerically higher proportions of triacylglycerol lipids containing 3, 4, 5, and 6 double bonds (17.06, 14.85, and 13.65% of triacylglycerol lipids with 3 or more double bonds in SF, LM, and IMF, respectively). While we cannot make this conclusion with certainty, these data may indicate that there is a preference for polyunsaturated fatty acids to be incorporated into triacylglycerol in SF rather than in IMF or LM. Across all tissues, TAG with 52 carbons and 2 double bonds was the most abundant. This observation is consistent with fatty acid composition of beef reported in the literature (Turk and Smith, 2009), likely representing two oleic acids and one palmitic acid. Turk and Smith (2009) reported that the linoleic acid content of eight different adipose tissue depots ranged from 1.63% to 1.98% using conventional fatty acid methodologies. Their study did not report other polyunsaturated fatty acids in those adipose tissue depots, likely because polyunsaturated fatty acids are typically found at low levels in beef due to ruminal biohydrogenation. The specificity of lipidomics may help resolve questions about where lipids that are present in smaller quantities, are deposited.

**Table 4.** Triacylglycerols in beef lean muscle, intramuscular fat, and subcutaneous fat^1^

Item	LM^2^	IMF^3^	SF^4^	SEM^5^	*P*-value
**Total carbons in acyl chains, mol % of triacylglycerols**					
**40**	0.05	0.06	0.04	0.008	0.23
**42**	0.11	0.13	0.12	0.013	0.60
**43**	0.02	0.04	0.02	0.016	0.48
**44**	0.46	0.50	0.45	0.045	0.64
**45**	0.11	0.16	0.14	0.021	0.36
**46**	1.90	2.01	1.86	0.204	0.87
**47**	0.56	0.70	0.69	0.077	0.37
**48**	8.11	8.00	7.72	0.548	0.87
**49**	1.87	2.11	2.19	0.170	0.42
**50**	19.5	18.8	18.4	0.67	0.50
**51**	3.93	4.05	4.48	0.279	0.36
**52**	42.2	40.7	39.0	1.01	0.11
**53**	3.38^a^	3.69^a,b^	4.27^b^	0.215	0.03
**54**	16.9	18.0	19.5	0.89	0.16
**55**	0.28^a^	0.35^b^	0.44^c^	0.019	<0.01
**56**	0.54^a^	0.54^a^	0.71^b^	0.031	<0.01
**58**	0.03	0.03	0.03	0.005	0.93
**Total double bonds in acyl chains, mol % of triacylglycerols**					
**0**	4.6	5.6	4.4	0.49	0.19
**1**	29.0^a^	34.0^b^	29.3^a^	1.46	0.05
**2**	51.5^a^	46.6^b^	49.2^a,b^	1.15	0.03
**3**	13.1	11.8	14.6	0.91	0.14
**4**	1.5	1.6	2.1	0.21	0.24
**5**	0.21	0.22	0.30	0.419	0.27
**6**	0.04	0.03	0.06	0.018	0.55

1 Within each row, means with differing superscripts differ (*P* ≤ 0.05).

2 LM, lean muscle.

3 IMF, intramuscular fat.

4 SF, subcutaneous fat.

5 SEM, standard error of the mean.

The three tissue types had distinct membrane lipid signatures. Membrane lipids in SF contained higher proportions of PC, PG, PS, and Cer than LM and IMF (*P *≤ 0.03; Table 5). Subcutaneous fat also contained a higher proportion of SM in membrane lipids than LM (*P *< 0.05) but was not different from IMF. Proportion of DAG in IMF and SF membrane lipids was higher compared to LM (*P *< 0.01). The distinct membrane lipid profiles found in LM, SF, and IMF may be explained by differences in activities of enzymes that convert one lipid class to another. For instance, LM contains less DAG as a proportion of membrane lipids than SF and IMF, but more PI as a proportion of membrane lipids than SF or IMF. The relationships of these lipids may be dependent upon difference in expression or activity of phospholipase C, an enzyme that converts PI to DAG, in LM and SF or IMF. Conversely, the abundance of DAG in SF and IMF may suggest that enzymes (lipin or diacylglycerol kinase) involved in the conversion of DAG to PA and vice versa may have reduced activity in SF and IMF. To our knowledge, the expression levels and activities of phospholipase C, lipin, and diacylglycerol kinase have not been compared in bovine LM, SF, and IMF either pre- or post-mortem, but the functions of these enzymes are well established in the literature (Bond, 2017).

**Table 5.** Membrane lipids in beef lean muscle, intramuscular fat, and subcutaneous fat^1^

Item	LM^2^	IMF^3^	SF^4^	SEM^5^	*P*-value
**Lipid Class, mol % of membrane lipids**					
**Cer^6^**	0.37^a^	0.45^a^	0.86^b^	0.107	0.01
**DAG^7^**	3.17^a^	10.47^b^	9.17^b^	0.762	<0.01
**PA^8^**	0.05^a^	0.09^a,b^	0.18^b^	0.052	0.01
**PC^9^**	34.28^a^	38.31^a^	41.85^b^	1.766	0.03
**PC O-^10^**	18.75^a^	11.04^b^	4.08^c^	1.116	<0.01
**PE^11^**	6.44	6.34	7.05	0.356	0.35
**PE O-^12^**	15.51^a^	10.23^b^	7.33^c^	0.537	<0.01
**PG^13^**	0.27^a^	0.23^a^	1.03^b^	0.081	<0.01
**PI^14^**	12.16^a^	7.46^b^	8.72^b^	0.829	<0.01
**PS^15^**	2.96^a^	4.65^a^	8.94^b^	0.893	<0.01
**SM^16^**	3.83^a^	9.20^a,b^	10.75^b^	1.609	0.02

1 Within each row, means with differing superscripts differ (*P* ≤ 0.05).

2 LM, lean muscle.

3 IMF, intramuscular fat.

4 SF, subcutaneous fat.

5 SEM, standard error of the mean.

6 Cer, ceramide.

7 DAG, diacylglycerol.

8 PA, phosphatidic acid.

9 PC, phosphatidylcholine.

10 PC O-, ether-linked phosphatidylcholine.

11 PE, phosphatidylethanolamine.

12 PE O-, ether-linked phosphatidylethanolamine.

13 PG, phosphatidylglycerol.

14 PI, phosphatidylinositol.

15 PS, phosphatidylserine.

16 SM, sphingomyelin.

Lean muscle contained higher proportions of ether-linked phospholipids than IMF and SF (*P *< 0.01). It is unclear why LM would contain more ether-linked lipids than the adipose tissues. In the biomedical literature, Donovan et al. (2013) demonstrated that obese human patients had higher levels of ether-linked lipids in plasma than lean patients. Although ether-linked lipids have been measured in bovine tissues, the role of ether-linked PC and PE in bovine skeletal muscle, particularly from fat/finished cattle, has not been characterized.

Phosphatidylcholine is the most abundant membrane lipid in LM, IMF, and SF (34.2–41.8 mol% of membrane lipids), and LM, IMF, and SF have distinct PC lipid compositions (Table 6). Lean muscle and IMF contain much higher proportions of PC 16:0_18:1 than SF whereas SF has higher proportions of longer-chain and more unsaturated PC species. Compared to LM, SF contained higher proportions of PC 16:0_20:3, PC 16:0_20:4, PC 16:1_18:0, PC 18:0_18:1, PC 18:0_18:2, PC 18:0_20:3, and PC 18:0_20:4 (*P *< 0.01). Similar to our observation with TAG, it appears that there is a biological preference for polyunsaturated fatty acids to be incorporated into SF rather than in IMF or LM. This finding could provide meaningful insight to those wanting to increase amounts of polyunsaturated fatty acids in beef for various nutritional benefits. Since the samples in this study were obtained from cattle fed a conventional finishing diet with the dietary fatty acids being subjected to ruminal biohydrogenation, we cannot draw conclusions about the preference for omega-3 fatty acids to be incorporated into SF over IMF or LM.

**Table 6.** Phosphatidylcholine species in lean muscle, intramuscular fat, and subcutaneous fat^1^

Item	LM^2^	IMF^3^	SF^4^	SEM^5^	*P*-value
**Lipid Species, mol % of PC^6^**					
**PC 16:0_16:0**	0.21^a^	1.27^b^	1.46^b^	0.299	0.01
**PC 16:0_16:1**	2.76	3.36	3.30	0.252	0.21
**PC 16:0_18:1**	40.91^a^	36.45^a^	24.42^b^	2.250	<0.01
**PC 16:0_18:2**	23.19^a^	17.04^a,b^	9.68^b^	2.270	<0.01
**PC 16:0_19:1**	0.54^a^	1.02^b^	1.03^b^	0.074	<0.01
**PC 16:0_20:3**	1.07^a^	1.98^a^	3.74^b^	0.427	<0.01
**PC 16:0_20:4**	1.95^a^	3.27^a,b^	4.91^b^	0.475	<0.01
**PC 16:0_22:5**	0.82	1.73	1.69	0.107	0.90
**PC 16:1_18:0**	0.40^a^	0.91^a^	2.06^b^	0.208	<0.01
**PC 16:1_18:1**	2.16	2.23	2.31	0.183	0.84
**PC 16:1_18:2**	1.29^a^	0.98^a,b^	0.73^b^	0.119	0.01
**PC 18:0_18:1**	3.78^a^	6.67^b^	8.69^b^	0.716	<0.01
**PC 18:0_18:2**	2.94^a^	3.86^a^	5.52^b^	0.289	<0.01
**PC 18:0_20:3**	0.16^a^	1.12^a^	3.13^b^	0.389	<0.01
**PC 18:0_20:4**	0.38^a^	1.56^a^	3.39^b^	0.364	<0.01
**PC 18:1_18:1**	4.53	4.45	4.01	0.335	0.51
**PC 18:1_18:2**	3.54	3.44	3.57	0.255	0.92

1 Within each row, means with differing superscripts differ (*P* ≤ 0.05).

2 LM, lean muscle.

3 IMF, intramuscular fat.

4 SF, subcutaneous fat.

5 SEM, standard error of the mean.

6 PC, phosphatidylcholine.

The membrane lipids in LM contained a greater proportion of PI than IMF or SF. For most of the PI species reported in Table 7, the greatest magnitude of difference was observed for PI 18:0_18:1, which represented 28 mol% of PI lipids in LM, but only 14–21 mol% of PI in SF or IMF, respectively (*P *< 0.01). Similar to what was observed in PC, SF generally contained higher proportions of polyunsaturated PI species. Compared to LM, SF contained higher proportions of PI 16:0_18:1, PI 16:0_20:3, PI 16:1_18:0, PI 18:0_20:3, PI 18:1_18:2, PI 18:1_20:3, and PI 18:1_20:4 (*P* ≤ 0.02). Interestingly, PI 18:0_20:4 was the most abundant PI species, and was not different, across the three tissues (27.8–29.1 mol%; *P *= 0.90).

**Table 7.** Phosphatidylinositol species in lean muscle, intramuscular fat, and subcutaneous fat^1^

Item	LM^2^	IMF^3^	SF^4^	SEM^5^	*P*-value
**Lipid Species, mol % of PI^6^**					
**PI 16:0_18:1**	0.58^a^	0.64^a^	1.40^b^	0.131	<0.01
**PI 16:0_20:3**	0.09^a^	0.38^a,b^	1.40^b^	0.447	0.02
**PI 16:1_18:0**	0.65^a^	2.36^b^	2.06^b^	0.317	<0.01
**PI 18:0_18:1**	28.51^a^	21.11^b^	14.21^c^	1.898	<0.01
**PI 18:0_18:2**	6.64	6.10	5.64	0.527	0.43
**PI 18:0_20:2**	1.78^a^	1.51^a^	0.62^b^	0.171	<0.01
**PI 18:0_20:3**	10.08^a^	12.78^a,b^	14.60^b^	0.846	<0.01
**PI 18:0_20:4**	29.07	27.88	29.16	2.268	0.90
**PI 18:0_20:5**	3.56^a^	3.01^a,b^	1.39^a^	0.575	0.03
**PI 18:0_22:3**	0.65^a^	7.96^b^	2.74^a,b^	2.324	0.03
**PI 18:0_22:4**	3.25^a^	7.87^b^	4.41^a,b^	1.159	0.03
**PI 18:0_22:5**	5.55^a^	5.49^a^	2.20^b^	0.525	<0.01
**PI 18:1_18:1**	2.32	2.29	3.11	0.268	0.08
**PI 18:1_18:2**	0.52^a^	0.65^a^	1.83^b^	0.232	<0.01
**PI 18:1_20:3**	0.65^a^	1.50^a^	3.38^b^	0.274	<0.01
**PI 18:1_20:4**	1.24^a^	2.78^b^	5.07^c^	0.276	<0.01

1 Within each row, means with differing superscripts differ (*P* ≤ 0.05).

2 LM, lean muscle.

3 IMF, intramuscular fat.

4 SF, subcutaneous fat.

5 SEM, standard error of the mean.

6 PI, phosphatidylinositol.

The adipose tissues, IMF and SF, contained much higher proportions of DAG than LM. This is likely because muscle is equipped with the cellular machinery to support contraction and oxidative metabolism rather than fat storage (El-Din et al., 2013). Thus, the DAG pool is lower in LM and DAG in LM may be more closely associated with cellular signaling and metabolic processes rather than serving as intermediates of triglyceride synthesis. Several DAG species were significantly enriched in either IMF or SF compared with LM, including DAG 16:0_16:1, DAG 16:0_18:0, DAG 18:0_18:1, and DAG 18:0_20:4 (*P* ≤ 0.04; Table 8). Significant differences in DAG 16:0_18:1, DAG 16:1_18:0, DAG 18:0_18:2, or DAG 18:1_18:1 were not observed (*P* ≥ 0.09) across LM, IMF, or SF. While IMF and SF did not differ in proportions of DAG 14:0_18:0 or DAG 14:0_18:1, SF contained more 14:0-containing DAG overall.

**Table 8.** Diacylglycerol species in lean muscle, intramuscular fat, and subcutaneous fat^1^

Item	LM^2^	IMF^3^	SF^4^	SEM^5^	*P*-value
**Lipid Species, mol % of DAG^6^**					
**DAG 14:0_18:0**	0.53^a^	1.33^a,b^	1.45^b^	0.266	0.01
**DAG 14:0_18:1**	2.47^a^	2.67^a,b^	3.90^b^	0.555	0.01
**DAG 16:0_16:1**	2.38^a^	3.41^b^	4.39^b^	0.318	<0.01
**DAG 16:0_18:0**	7.93^a^	16.28^b^	11.73^a,b^	2.050	0.04
**DAG 16:0_18:1**	25.20	35.10	27.87	3.322	0.13
**DAG 16:1_18:0**	1.06	1.66	1.12	0.210	0.12
**DAG 18:0_18:1**	17.31^a^	22.53^b^	15.52^a^	1.377	<0.01
**DAG 18:0_18:2**	2.93	3.47	2.00	0.44	0.09
**DAG 18:0_20:4**	2.14^a^	13.26^b^	10.14^b^	1.29	<0.01
**DAG 18:1_18:1**	21.46	15.85	17.51	1.77	0.11

1 Within each row, means with differing superscripts differ (*P* ≤ 0.05).

2 LM, lean muscle.

3 IMF, intramuscular fat.

4 SF, subcutaneous fat.

5 SEM, standard error of the mean.

6 DAG, diacylglycerol.

Lysolipids make up ≤1% of total lipids in LM, IMF, and SF, with LPC being the most abundant lysolipid across all tissue types, accounting for 41.6–55.4 mol% of total lysolipids. Intramuscular fat contained a greater proportion of LPC compared to LM and SF (*P *< 0.01; Table 9). Lysophosphatidylethanolamine (LPE) comprised approximately 20–30 mol% of total lysolipids and was slightly higher in IMF than LM and SF but was not significantly different across tissue types (*P *< 0.08). Lean muscle contained fewer storage lipids and more membrane and lysolipids than IMF and SF; interestingly, LM also contained greater proportions of LPC O- and LPE O- than IMF or SF (*P *< 0.01). The relationship between ether-linked phospholipids (including plasmalogens), which act as antioxidants by scavenging reactive oxygen species, and shelf life and lipid stability of meat is largely unknown. Lysophosphatidylinositol (LPI) content was highest in SF, intermediate in IMF, and lowest in LM (*P *< 0.01). There was no difference in proportion of LPA, lysophosphatidylglycerol (LPG), or lysophosphatidylserine (LPS) in LM, IMF, or SF (*P *> 0.08).

**Table 9.** Lysolipids in beef lean muscle, intramuscular fat, and subcutaneous fat^1^

Item	LM^2^	IMF^3^	SF^4^	SEM^5^	*P*-value
**Lipid Class, mol % of lysolipids**					
**LPA^6^**	0.99	2.56	1.86	0.707	0.24
**LPC^7^**	41.60^a^	55.41^b^	47.15^a^	1.912	<0.01
**LPC O-^8^**	7.23^a^	2.61^b^	1.72^b^	0.591	<0.01
**LPE^9^**	25.68	29.15	21.91	2.044	0.08
**LPE O-^10^**	16.69^a^	6.39^b^	5.94^b^	1.023	<0.01
**LPI^11^**	3.78^a^	9.06^a, b^	18.37^b^	4.917	<0.01
**LPG^12^**	0.12	0.45	0.56	0.258	0.36
**LPS^13^**	3.98	3.30	2.45	0.435	0.08

1 Within each row, means with differing superscripts differ (*P* ≤ 0.05).

2 LM, lean muscle.

3 IMF, intramuscular fat.

4 SF, subcutaneous fat.

5 SEM, standard error of the mean.

6 LPA, lysophosphatidic acid.

7 LPC, lysophosphatidylcholine.

8 LPC O-, ether-linked lysophosphatidylcholine.

9 LPE, lysophosphatidylethanolamine.

10 LPA O-, ether-linked lysophosphatidylethanolamine.

11 LPI, lysophosphatidylinositol.

12 LPG, lysophosphatidylglycerol

13 LPS, lysophosphatidylserine.

We did not measure the pH of the loins in this study because it was outside of the scope of this article; however, all loins were obtained from carcasses that qualified for the Certified Angus Beef branded program and did not have signs of discoloration in the commercial packing facility or in our laboratory at the time of sample collection. However, it may be worthwhile to consider how changes in pH or discoloration may impact the lipidome and the whole food beef matrix.

## Conclusions

Lean muscle, intramuscular fat, and subcutaneous fat each contribute different lipids to the whole food beef matrix. In future investigations concerning the impact of beef consumption on human health, it will be important to consider the proportions of lean and fat in beef products that are consumed. Additional research should be conducted to define the lipidome of other beef retail cuts. Further, additional research should be conducted to determine how beef aging, packaging, further processing, and cooking methods impact the lipidome and other properties of the whole food beef matrix.

## Abbreviations

CL: cardiolipins
Cer: ceramide
DAG: diacylglycerol
HexCer: hexosylceramide
LPA: lysophosphatidic acid
LPC: lysophosphatidylcholine
LPC O-: ether-linked lysophosphatidylcholine
LPE: lysophosphatidylethanolamine
LPE O-: ether-linked lysophosphatidylethanolamine
LPG: lysophosphatidylglycerol
LPI: lysophosphatidylinositol
LPS: lysophosphatidylserine
PA: phosphatidic acid
PC: phosphatidylcholine
PC O-: ether-linked phosphatidylcholine
PE: phosphatidylethanolamine
PE O-: ether-linked phosphatidylethanolamine
PG: phosphatidylglycerol
PI: phosphatidylinositol
PS: phosphatidylserine
SM: sphingomyelin
TAG: triacylglycerol
